# Supplementary material for: An Investigation of Burkholderia pseudomallei Seroprevalence in Market Pigs Slaughtered at Selected Pig Abattoirs in Uganda
Source: Pathogens. 2022 Nov 16;11(11):1363. doi: 10.3390/pathogens11111363 (PMC9699407; doi:10.3390/pathogens11111363)
Supplement: Supplementary file 1 [file pathogens-11-01363-s001.zip › pathogens-2000769-supplementary.pdf]

Table S1: Temporal distribution of *Burkholderia pseudomallei* seroprevalence in market pigs slaughtered at selected pig abattoirs in Uganda

Month	Two standard deviation cutoff						Three standard deviation cutoff						Total
	**# positive A**	**% (95%CI) positive A**	**# positive B**	**% (95%CI) positive B**	**# positive both A & B**	**% (95%CI) positive both A & B**	**# positive A**	**% (95%CI) positive A**	**# positive B**	**% (95%CI) positive B**	**# positive both A & B**	**% (95%CI) positive both A & B**	
Jan	2	2.00% (0.1-7.4%)	2	2.00% (0.1-7.4%)	2	2.00% (0.1-7.4%)	0	0.00%	0	0.00%	0	0.00%	100
Feb	0	0.00%	1	0.93% (0-5.6%)	0	0.00%	0	0.00%	0	0.00%	0	0.00%	108
Mar	1	0.98% (0-5.9%)	2	1.96% (0.1-7.3%)	0	0.00%	0	0.00%	1	0.98% (0-5.9%)	0	0.00%	102
Apr	1	4.00% (0-21.1%)	2	8.00% (1.1-26.1)	0	0.00%	0	0.00%	0	0.00%	0	0.00%	25
May	0	0.00%	1	4.00% (0-21.1%)	0	0.00%	0	0.00%	0	0.00%	0	0.00%	25
Jun	0	0.00%	2	3.08% (0.2-11.2%)	0	0.00%	0	0.00%	0	0.00%	0	0.00%	65
Jul	0	0.00%	2	2.13% (0.1-7.9%)	0	0.00%	0	0.00%	1	1.06% (0-6.4%)	0	0.00%	94
Aug	0	0.00%	0	0.00%	0	0.00%	0	0.00%	0	0.00%	0	0.00%	104
Sep	1	0.95% (0-5.7%)	3	2.86% (0.6-8.4%)	0	0.00%	0	0.00%	0	0.00%	0	0.00%	105
Oct	19	18.10% (11.8-26.6%)	20	19.05% (12.6-27.7%)	10	9.52% (5.1-16.8%)	4	3.81% (1.2-9.7%)	6	5.71% (2.4-12.2%)	0	0.00%	105
Nov	23	23.47% (16.1-32.8%)	34	34.69% (26-44.6%)	15	15.31% (9.4-23.8%)	4	4.08% (1.3-10.4%)	12	12.24% (7-20.3%)	0	0.00%	98
Dec	24	23.08% (16-32.1%)	24	23.08% (16-32.1%)	17	16.35% (10.4-24.7%)	12	11.54% (6.6-19.2%)	8	7.69% (3.7-14.7%)	0	0.00%	104

# = number.
